# Sol-gel synthesis of amorphous calcium phosphate nanoparticles in brown rice substrate and assessment of their cytotoxicity and antimicrobial activities 

**DOI:** 10.22038/AJP.2021.18930

**Published:** 2022

**Authors:** Sima Beigoli, Azadeh Hekmat, Fahimeh Farzanegan, Majid Darroudi

**Affiliations:** 1 *Department of Biology, Science and Research Branch, Islamic Azad University, Tehran, Iran *; 2 *Department of Orthodontics, Oral & Maxillofacial Diseases Research Center, School of Dentistry, Mashhad University of Medical Sciences, Mashhad, Iran *; 3 *Nuclear Medicine Research Center, Mashhad University of Medical Sciences, Mashhad, Iran*; 4 *Applied Biomedical Research Center, Mashhad University of Medical Sciences, Mashhad, Iran*; 5 *Department of Medical Biotechnology and Nanotechnology, School of Medicine, Mashhad University of Medical Sciences, Mashhad, Iran*

**Keywords:** Sol-gel synthesis, Amorphous calcium phosphate (ACP), Nano-biomaterials, Antibacterial activity Cytotoxicity

## Abstract

**Objective:**

This study intended to perform a synthesizing procedure for amorphous calcium phosphate (ACP) through a green template by the usage of brown rice (BR).

**Materials and Methods:**

ACP nanoparticles were obtained by application of a sol-gel method and comprehensively characterized using X-ray powder diffraction (XRD), zeta potential, fourier-transform infrared spectroscopy (FTIR), field emission scanning electron microscope (FESEM), and atomic force microscopy (AFM). Cytotoxic activity of ACP was evaluated in human epithelial type 2 (HEp-2) cell lines. The antibacterial effects of nanoparticles were appraised against Gram-positive *Streptococcus mutans* and *Enterococcus faecalis*.

**Results:**

The procedures for the evaluation of the characterization outcomes, dispersion, and stability of our product were confirmed by observing the smooth and uniformed surfaces of ACP. The zeta potential value of the synthesized sample was -22 mV, which indicates its acceptable stable condition caused by electrostatic repulsion. The cytotoxicity of the ACP nanoparticles was investigated in HEp-2 cells, and results showed no cytotoxicity for the synthesized nanoparticles. Also, the obtained minimum inhibitory concentration (MIC) of ACP nanoparticles in opposition to *S. mutans and E. faecalis* was 15 and 20 µg/ml, respectively, indicating the resistance of *E. faecalis* in comparison to *S. mutans* and MBC for synthesized nanoparticles against *S. mutans and E. faecalis *strains was 20 and 25 µg/ml.

**Conclusion:**

The present study showed that this compound has no toxicity on the examined cell line. Also, the antibacterial properties of the synthesized ACP were approved by the obtained data, which enables the application of this material for therapeutic purposes in the pharmaceutical industry.

## Introduction

Amorphous calcium phosphate (ACP) is a supersaturated solution of solid calcium phosphate particles that contains the crystalline products of octa-calcium phosphate (OCP) with the vital responsibility of acting as the precursor of bioapatite, as well as functioning as a transitional phase in the process of biomineralization (Ikawa et al., 2009 ▶; Karimi et al., 2016 ▶; Somrani et al., 2003 ▶). There is a vast range of ACP implementations throughout the fields of medicine, water treatment, material science, and biology, due to offering a list of notable features such as high surface to volume ratio, lack of toxicity, lack of inflammatory signs, osteointegrity, being stable throughout neutral and basic conditions, lack of immunogenicity, biocompatibility, bioactivity, low water solubility in an acidic environment, osteoconductivity, and fracture toughness (Gopi et al., 2012 ▶). The amazing solubility of this product is provided by its amorphous construction, hydrated layer, and defects (Sondi and Salopek-Sondi, 2004 ▶). To be explained in detail, the fabrication of structural defects is facilitated by the lack of periodic long-range order, which results in intensifying the rates of solubility and resorption that consequently enhance the bioactivity of ACP. Most importantly, due to being considered a biological agent, ACP is widely used for bone repairing/ tooth defects, implants and gene delivery/drug delivery, and tissue engineering (Vecstaudza et al., 2019 ▶). Despite these facts, the combination of temperature, suitable nutrition, and moisture of the human body with the biological activity of ACP helps the existing bacteria to multiply on the implant surfaces, resulting in the occurrence of severe physiological damages and implant failure (Phatai et al., 2019 ▶). Thus, the necessity to evaluate the antibacterial functionality and cytotoxicity of ACP, as well as its altered forms, for preventing the need for additional medical procedures is quite evident.

Throughout the recent decade, the exertion of varying procedures has been reported for the production of ACP including microemulsion technique, sol-gel, incipient wet chemical route, chemical precipitation, solid-state reaction, and mechanochemical procedure (Khan et al., 2021 ▶; Phatai et al., 2019 ▶). However, the sol–gel routes offers certain benefits since the fabricated ACP particles are reported to contain nano-sized dimensions, stoichiometric construction, high purity, and enhanced surface area. In the past years, the development of hydroxyapatite (HAp) by combining template addition with any synthesizing method proved to be applicable for fabricating distinctive nanoparticles that would accommodate a homogenous morphology, narrow particle size distribution, and minimal particle aggregation (Gopi et al., 2013 ▶; Khan et al., 2021 ▶). 

Brown rice (BR) is an essential staple food that grows under flooded conditions. Irrigation of this crop with As-contaminated water leads to the accumulation of toxins in different parts of the plant tissues, which would be subsequently transported to the human food chain. Nowadays, various methods, such as physical, chemical, and biological procedures, are practiced to remediate polluted water (Shafie and Esa, 2017 ▶). Physical and chemical techniques are not suitable for long runs due to being costly, lower in efficacy, and less environmentally friendly, whereas there are varying “functional groups” that naturally exist on the surface of biologically synthesized nanoparticles (Banerjee et al., 2013 ▶; Khan et al., 2021 ▶; Lim et al., 2014 ▶). Therefore, many scientists focused on synthesizing metal nanoparticles by utilization of biological procedures due to containing certain qualities such as low toxicity, bio-compatibility, and environmentally friendly manner (Khan et al., 2021 ▶). The widespread usage of BR as an industrial source is associated with the existing high volume of amylopectin that accommodates a unique crystalline arrangement consisted of tandem-linked clusters (Patil and Khan, 2011 ▶). Based on extensive literature review, there are no reported studies on the antibacterial activity of ACP particles prepared by the technique of sol-gel procedure combined with the green template addition method using BR (Shafie and Esa, 2017 ▶). In this work, we succeeded in performing the synthesis of ACP through a sol-gel procedure that involved the extraction of BR as a green template and also, we investigated the physicochemical properties of the obtained product. Its antibacterial qualities were also evaluated in opposition to the applied bacteria. In addition, the cytotoxicity of this product in Human epithelial type 2 (HEp-2) cells, which is available in the oral cavity, was examined.˚

## Materials and Methods

**Materials**

Trisodium phosphate (Na_3_PO_4_) and calcium chloride (CaCl_2_) were obtained from Sigma-Aldrich. Boiling dried BR seeds were also prepared and exerted to initiate the upcoming procedure. The utilized Hep-2 (Human epithelial type 2, human laryngeal carcinoma) cells were purchased from the Pasteur Institute cell bank in Tehran, Iran. RPMI-1640 and DMEM (Biosera-UK) medium were equipped with 10% of fetal calf serum, 1% of penicillin, and 1% of streptomycin (Biosera-UK). Finally, the cell cultures were grown by the usage of a 5% CO_2_ incubator at the temperature of 37°C. BuAli Research Institute of Mashhad, Iran, supplied our experimental bacteria that involved *Streptococcus mutans* (ATCC 35668) and *Enterococcus faecalis* (ATCC 29212), which were applied as the subculture in 5% sheep's blood agar.

**Synthesis of ACP nanoparticles**

**Figure 1:**
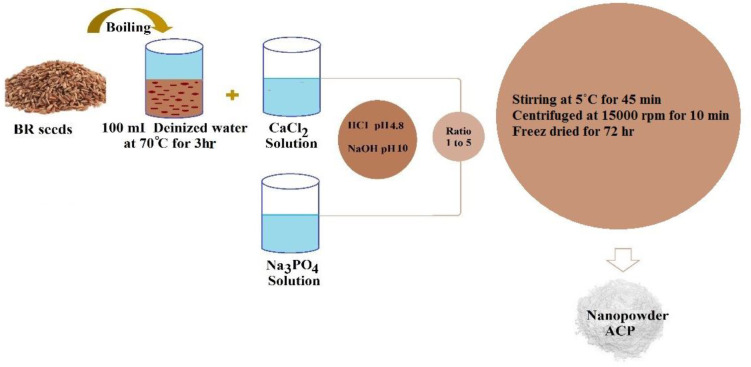
The schematic plan synthesis process of amorphous calcium phosphate (ACP) nanoparticles

A sol-gel procedure was exerted to synthesize ACP with the usage of BR as a template. To formulate the template solution, the dried BR seeds (4.0 g) were boiled within 100 ml of deionized water at the temperature of 70°C for 3 hr. Thereafter, the mixture of dissolved CaCl_2_ solution in 50 ml of deionized water was appended to the boiling dried BR seeds to prepare the required solution (0.1 M), which was kept overnight at 5 °C. After addition of Na_3_PO_4_ (2.527 g) to 50 ml of deionized water, the obtained solution was mixed with CaCl_2_ and boiling dried BR seeds solutions in a ratio of 1:5 to go through a stirring process for 45 min at 5°C. Once the mixture was repeatedly incubated, the produced product was centrifuged at 15000 rpm for 10 min. We reran this procedure several times to detach the sodium and chlorine ions; as the next step, the sediment was freeze-dried for 72 hr. Figure 1 displays the schematic plan of ACP nanopowders synthesizing procedure. 

**Characterization**

Assessment of crystal construction, size of particles, morphology, chemical composition, and configuration of functional groups of ACP samples was performed through varying methods. These procedures included X-ray diffraction (XRD, Siemens D-500 diffractometer, the data were obtained through a step size at 0.02 s^-1^ and a scanning range of 2θ=10 to 70°C, field-emission scanning electron microscopy (FESEM, Tescan Mira 3 LMU), energy-dispersive X-ray spectroscopy (EDS, Bruker, Quantax 200), Fourier transform infrared spectroscopy (FTIR, PerkinElmer Spectrum 400, range 400-4000 cm^−1^ with a resolution of 4 cm^−1^) and Atomic Force Microscopy (AFM, Nanosurf®Mobile S., Switzerland) that exhibits the distribution and average diameter of nanoparticle, as well as Brunauer–Emmett–Teller (BET) N_2_ adsorption analysis for assessing SSA with Quadrasorb SI (Quantachrome) unit; the samples were ascertained to be degassed at ambient temperature for 24 hr before going through the aforementioned measurements.

**Antibacterial test**

We evaluated the antibacterial activities of the produced ACP nanoparticles through application of agar well diffusion and microdilution techniques in opposition to two bacterial strains *Streptococcus mutans* (ATCC 35668) and *Enterococcus faecalis* (ATCC 29212). Different concentrations of the sample were prepared and sterilized by Müller-Hinton broth culture medium. Finally, a certain volume of bacteria in physiological serum was added to each sample in a way that the number of bacteria would be equated to 100,000 bacteria per milliliter, which were placed in an incubator at a temperature of 37°C. To complete our data, we prepared a positive control group (culture medium with bacteria) and a negative control group (untreated, solvent). After 24 hr, the minimum inhibitory concentration (MIC) of growth was configured through a color reduction method. The exerted color was resazurin, which implies cell viability through the alteration of color from a blue/non-T fluorescent state to a pink/highly fluorescent state through a chemical reduction caused by aerobic respiration due to cell growth. Color changes are visually inspected to determine the growth of minimum inhibitory concentration. The lowest concentration without displaying any color changes, also expressed as the lowest concentration that prevented the occurrence of any growth, is reported as the MIC.

**Cytotoxicity assay**

The cytotoxicity of ACP nanoparticles was assessed by exerting the MTT assay. In brief, the designated HEP-2 cells (5×10^3^ cells) were seeded within the wells of a 96-well plate to be incubated for the duration of 24 hr at 37°C while being supplied with 5% CO_2_. In the following, we exposed the cells to various concentrations (31.25, 62.5, 125, 250, 500 and1000 μg/ mL) of ACP nanoparticles, which were allowed to grow for another 48 hr. After treating each well with methyl thiazolyl tetrazolium (MTT) for a period of 4 hr, the cell viability was calculated through a Microplate Reader at 550 nm in conformity to the absorbance of liquified formazan crystals within dimethyl sulfoxide (DMSO).

## Results

**Zeta potential and dynamic light scattering (DLS)**

**Figure 2:**
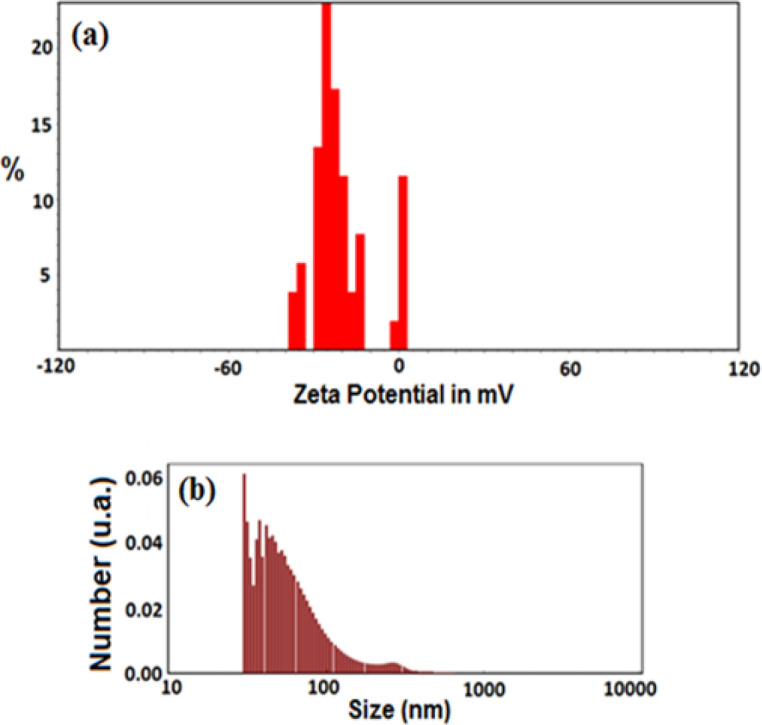
The Zeta potential (a) and particle size distribution (b) of amorphous calcium phosphate (ACP) nanoparticles

The measurements of surface charges and particle size were performed by the means of a Zeta-sizer Nano series (Malvern Instrument, Royston, UK) and dynamic light scattering (DLS) method, respectively. As it is known, certain fundamental data on the stability of a colloid system can be attained through Zeta potential assessment (Zhang et al., 2008 ▶). The value of Zeta (ξ) potential refers to the available electrostatic potential of the shear plane of a particle that is attributed to the surface charge and local environment of the particle (Hunter, 2013 ▶; Zhang et al., 2008 ▶). The obtained outcomes of Zeta potential measurements at pH 7.4. was displayed in Figure 2a. According to the results, the Zeta potential value of the synthesized sample was -22 mV, which exhibited the negative zeta potential of this product and indicated its acceptable stable condition caused by electrostatic repulsion. In addition, the average size of the synthesized sample (in aqueous solution) as measured by DLS (Figure 2b), was 329±66 nm. Polydispersity index (PDI) of monodisperse ACP nanoparticles was 0.28 and the measurement below 0.5 indicated the presence of monodispersity particles.

**FTIR spectroscopy**

**Figure 3:**
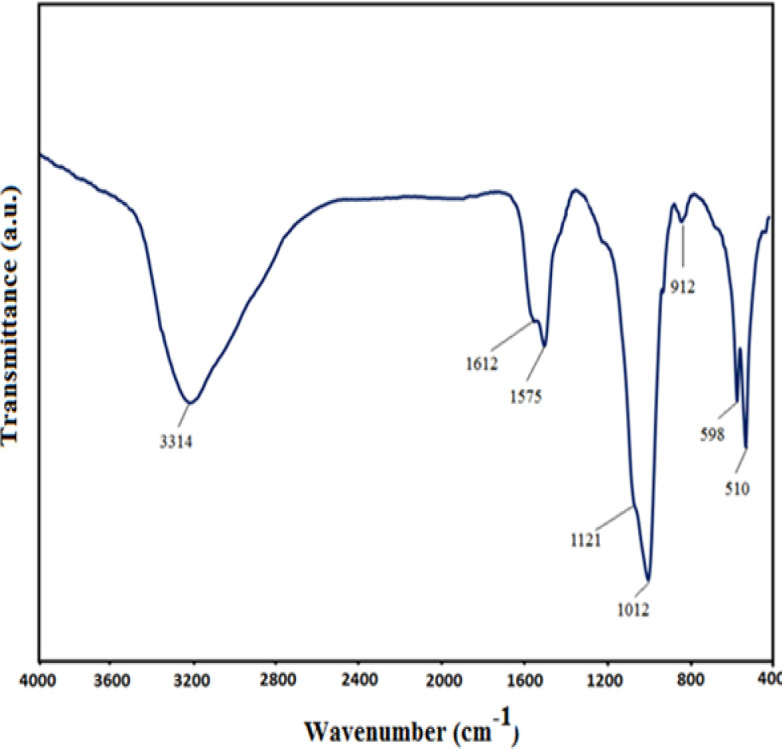
The Fourier-transform infrared spectroscopy (FTIR) spectra of amorphous calcium phosphate (ACP) nanoparticles

**C**onsidering FT-IR spectra of ACP nanoparticles in Figure 3, the broad absorption peaks observed at 3314, 1612, and 1575 cm^−1^ are associated with the –OH group of water molecules (Huang et al., 2017 ▶; Ibsen et al., 2016 ▶). Furthermore, the absorption detected bands at 1121 and 912 cm^−1^ was in correlation to the P-O stretching vibration bands of P-O, while the other two bands at 598 and 510 cm^−1^ were caused by the bending vibration band of P-O that stands as the signs of PO_4_^3−^ ions bands. We also perceive a single band at 598 cm^−1^ could refer to the products of ACP; however, it is assumed that the anisotropic local electric field of crystalline apatite becomes divided into an apparent doublet absorption band between 500 and 600 cm^−1.^ Lastly, the existence of ACP molecules led to the inducement of an intense absorption band at about 1121 cm^−1^ (Brangule and Gross, 2015 ▶; Sabouri et al., 2019 ▶); these observations are comparable to the outcomes of XRD. 

**XRD pattern**

**Figure 4:**
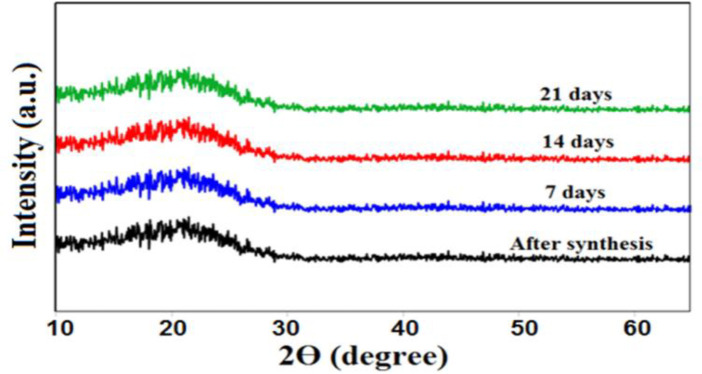
The X-ray powder diffraction (XRD) pattern of ACP nanoparticles

We were able to examine the crystalline construction of our samples by the application of the XRD method. The storage stability of ACP nanoparticles was assessed by placing the samples at room temperature for 21 days and distinguished by XRD at varying time intervals. In conformity to Figure 4, next to the lack of detecting any diffraction peaks, the existing broad and curve bread peaks at around 2θ= 22°C are suggestive of the amorphous phase of synthesized particles after being stored for 21 days in the air. A higher solubility and reactivity of amorphous structures, in comparison to that of the crystalline structures, was confirmed by the obtained outcomes, which can lead to intensifying the speed of the apatite formation process and increasing the bioactivity and biocompatibility features (Chahkandi et al., 2019 ▶; Chahkandi and Mirzaei, 2017 ▶; Li et al., 2007 ▶; Niu et al., 2020 ▶). 

**FESEM/EDAX images**

**Figure 5:**
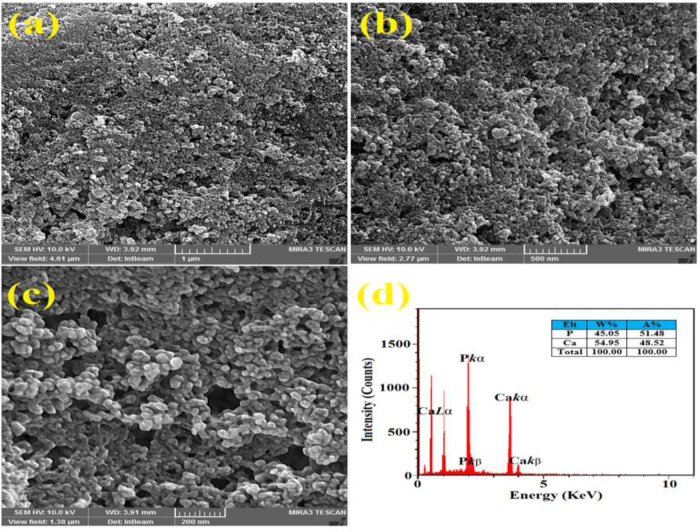
The field emission scanning electron microscope (FESEM) images of ACP nanoparticles at various scales (a-b) and energy dispersive X-ray (EDAX) analyze (c)

Measurement (diameter, nanoscale), morphology, and structure of the synthesized ACP sample were investigated by FESEM. As it can be observed in Figure 5 (a, b and c), many particles are agglomerated and the rest are displayed as separate grains. In conformity to the obtained diffraction pattern, the studied powder represents a non-related substance to the "crystalline" phase. The recognizable points of this image indicate the formation of a nanometer-shaped structure. The presence of Ca and P can be observed throughout the EDAX results (Figure 5d) (Čadež et al., 2018 ▶).

**Atomic force microscopy (AFM)**

**Figure 6:**
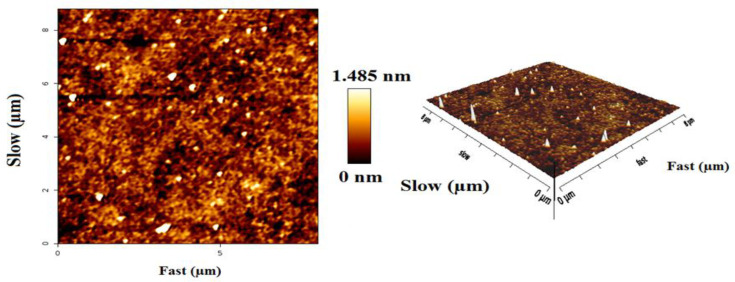
Atomic force microscopy (AFM) images of ACP nanoparticles

The Atomic force microscopy (AFM) image was applied to analyze the distribution and average diameter of nanoparticles. The analysis conducted by the usage of AFM images helped in configuring the morphology and size range of nanometer-sized particles adsorbed on flat surfaces (Philip and Walsh, 2019 ▶). Furthermore, the existence of a smooth and uniformed surface was evident throughout the outcomes, which was caused by the reduced particle size of the sample (Figure 6). 

**The antibacterial assessment of ACP**

**Figure 7:**
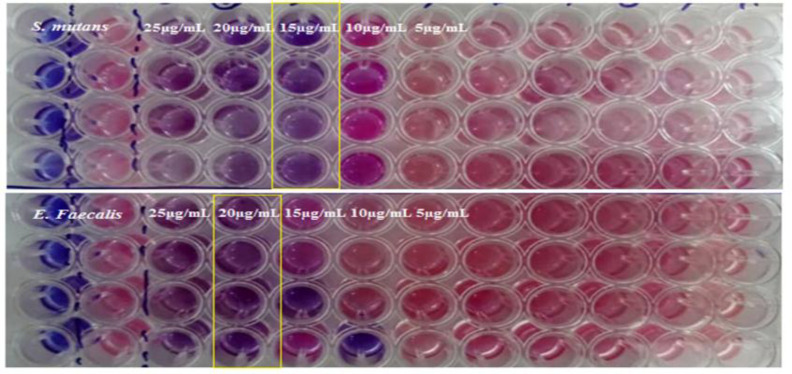
MIC of ACP nanoparticles against *S. mutans* (a) and *E. faecalis* (b) strains on resazurin microtiter plate assay (REMA) method was 15 and 20 µg/ml. Serial five-fold dilutions of ACP nanoparticles at 25, 20, 15, 10, 5 and 0 µg/ml

The MIC results of ACP were indicative of its antibacterial functionality in opposition to *S. mutans and E. faecalis *(Figure 7 a and b). In this study, the obtained MIC of ACP nanoparticles against *S. mutans and E. faecalis* was 15 and 20 µg/ml, respectively, which represents the resistance of *E. faecalis *in comparison to *S. mutans* toward the synthesized ACP. Also, The MBC of synthesized nanoparticles against *S. mutans and E. faecalis *strains was 20 and 25 µg/ml (Table 1). The mechanism of ACP antibacterial activity, as reported in previous studies, is mediated via destroying the stability of cytoplasmic membranes by creating a pore or targeting intracellular molecules and disrupting protein synthesis, DNA, enzyme activity, or the cell wall, which leads to the destruction of target cells (Matinfar et al., 2019 ▶; Philip and Walsh, 2019 ▶). As it was proven by the results of this work, the durability and antibacterial potency of ACP can be increased by doping certain bioactive materials, such as peptides isolated from casein micelles, which would lead to intensifying the activity of peptides-ACP complexes by being incorporated into ACP (Rodino et al., 2015 ▶). 

**Table 1:** Minimum bactericidal concentration (MBC) and Minimum inhibitory concentration (MIC) of ACP nanoparticles against oral bacteria (µg/ml).

Strains	*Streptococcus mutans*		*Enterococcus faecalis*	
	MBC	MIC	MBC	MIC
Chlorhexidine (0.2%)	25	25	25	25
ACP	20	15	25	20

**Cytotoxicity assay**

**Figure 8:**
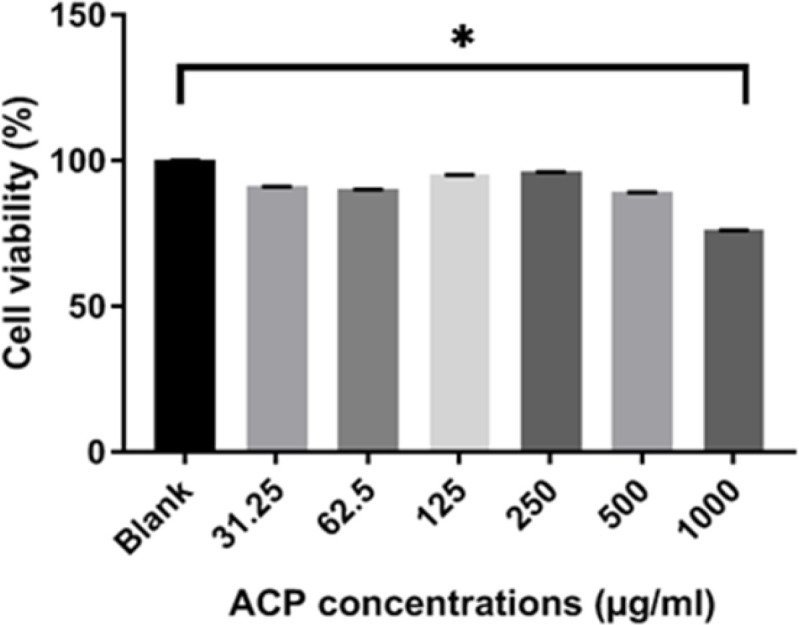
Cytotoxic effects of ACP nanoparticles in cultured HEp-2 cells at 31.25, 62.5, 125, 250, 500 and 1000 µg/ml after 48 hr treatment. Data are reported as the mean±SEM.*p<0.05 shows significant differences compared to the control

We performed the MTT assay on HEp-2 cells to evaluate and examine the cytotoxicity of the obtained ACP, and according to observations, this product could suppress the growth of cancer cells (Figure 8). Cell viability was close to 100% at different concentrations of ACP while no toxicity on HEp-2 cells was observed. Additionally, a notable difference was observed between the control group and the cells treated with 1000 µg/ml of ACP after 48 hr. Also, the cytotoxicity of ACP against HEp-2  cell line has not exhibited any cytotoxic effects (Kamelnia et al., 2020 ▶; Sabouri et al., 2020 ▶). 

## Discussion

In this work, we succeeded in conducting the synthesis of ACP through a sol-gel procedure that involved the extraction of BR as a green template and investigated the physicochemical and antibacterial properties of the obtained product. In addition, we examined the cytotoxicity of this product in HEp-2 cells, which are available in the oral cavity. 

Electro synthetic potential or zeta potential is the potential difference between the last solution layer containing the colloidal particles and the first immobile layer of solvent around the colloidal particles (Clogston and Patri, 2011 ▶). This value indicates the amount of repulsion between adjacent particles (Salopek et al., 1992 ▶). The measured potential of ACP was -22 mV, which is approximately similar to that reported in previous studies (Chen et al., 2014 ▶; Varasteh et al., 2019 ▶). Therefore, these results can be useful for understanding the mode of interaction that occurs in biological systems. Previous studies reported that the XRD peak broadening of a sample is considered a sign of amorphous phase (Elgamily et al., 2019 ▶). In addition, the results obtained for the synthesized ACP displayed an X-ray diffraction pattern in the form of a single broad peak. It is noteworthy that the provided data by X-ray diffraction lines is an available was of detecting+ nanometer materials (Kumar and Singh, 2015 ▶). In this research, the FTIR pattern of the synthesized ACP exhibited a peak at 510 cm^-1^ that related to the vibrations of bending group P-O, which is a characteristic of PO_4_^3−^ ions, and proves the amorphous structure of calcium phosphate products. This observation is similar to the results of Akgul et al. research and stands as the first report of ACP production (Akgul and Kaya, 2004 ▶). In conformity to the outcomes of Field Emission Scanning Electron Microscope (FESEM), the obtained ACP nanoparticles emerged in the form of agglomerates, while some of them appeared as separate granules; these results are consistent with the data reported by other researchers (Niu et al., 2020 ▶).

An overview of the findings of this study shows non-toxicity of the synthesized compounds that were achieved through a sol-gel procedure and therefore, they can be suggested as an appropriate candidate for being applied in different biomedical applications (Beigoli S, 2021 ▶). Furthermore, another study by Simon Jr and colleagues assessed the cytotoxicity of ACP in MC3T3-E1 osteoblast-like cells using MTT assay and confirmed the non-toxic behavior of this compound on the experimented cells (Simon Jr et al., 2005 ▶).

In the course of the polymerization, shrinkage is recognized as one of the defects of composites that leads to the inducement of a gap between the edges of tooth and restoration, which can cause bacterial leakage and secondary decay in long run as well (Chahkandi et al., 2019 ▶). As a result, many scientists attempted to take the challenge of creating antimicrobial properties by mixing composites with other materials. In this regard, this study aimed to mix ACP nanoparticles with composites to prevent the growth of *Streptococcus mutans* and *Enterococcus faecalis*, which are known as one of the most vital causes of tooth decay. In a similar study, mesoporous calcium-silicate nanoparticles loaded with chlorhexidine exhibited the release of ions and chlorhexidine, low cytotoxicity, excellent antibacterial ability, and *in vitro* mineralization. This sample could be developed into a new effective intra-canal medication product in dentistry or orthopedics as a novel bone defect filling material for infected bone defects, which would be consistent with this study (Fan et al., 2016 ▶). Sondi and Salopek-Sondi, reported the antimicrobial activity of silver nanoparticles on Gram-positive bacteria (Sondi and Salopek-Sondi, 2004 ▶). In this study, the antibacterial activity of the synthesized compound was compared with the other plant compounds and also, we experimented with the synthesized nanoparticles in the cases of two bacterial classes. According to the outcomes, the synthesized nanoparticles exhibited relatively stronger antibacterial effects on the *Enterococcus faecalis* bacterial class when being compared to the other studied compounds by other researchers. Nevertheless, the level of antibacterial activity in the extracted solution and synthesized nanoparticles was observed to be concentration-dependent (Akgul and Kaya, 2004 ▶; Elgamily et al., 2019 ▶; Kumar and Singh, 2015 ▶; TURU et al., 2020 ▶; Varasteh et al., 2019 ▶). Due to their smaller size, ACP nanoparticles have a higher level of contact with the environment and microorganisms. This feature increases their biological and chemical activities, which consequently enable them to create a greater impact on cell membranes. Therefore, we can suggest the stance of nanoparticles as a next-generation antibacterial agent for being applied in various biomedical applications (Chahkandi and Mirzaei, 2017 ▶).

We successfully prepared ACP nanoparticles by the usage of rice seedlings via the described sol-gel technique. The structure, morphology, and composition of obtained ACP nanoparticles were thoroughly distinguished by applying FESEM and EDS measurements, while their amorphous structure was confirmed via FTIR and XRD measurements. In conformity to the MTT results, the synthesized nanoparticles did not cause any cytotoxicity on the experimented cell line. Moreover, the antibacterial properties of the synthesized ACP were proved by the antibacterial assessments, which makes it viable as a cost-effective and available source for therapeutic applications in oral health. 

In summary, our results provided a facile approach for producing ACP nanoparticles with relatively narrow size distributions through a sol-gel method, improving their stability in preserving the amorphous phase. 

## Conflicts of interest

The authors have declared that there is no conflict of interest.
